# IL-1RN VNTR, IL-2(-330), and IL-4 VNTR gene polymorphisms in patients with chronic rhinosinusitis with sinonasal polyposis

**DOI:** 10.3906/sag-1710-186

**Published:** 2019-10-24

**Authors:** Gökhan KURAN, Hüseyin ASLAN, Süheyl HAYTOĞLU, Özge ÖZALP YÜREĞİR, Sevcan TUĞ BOZDOĞAN

**Affiliations:** 1 Adana City Training and Research Hospital, ENT Department, Adana, Turkey; 2 Assistant Professor, Eskisehir Osmangazi University Medical Faculty, Department of Medical Genetics, Eskisehir Turkey; 3 Associate Professor, Adana City Training and Research Hospital, Department of Medical Genetics, Adana Turkey; 4 Assistant Professor, Mersin University Medical Faculty, Department of Medical Genetics, Mersin Turkey

**Keywords:** Nasal polyposis, IL-1RN VNTR, IL-2(-330), IL-4 VNTR, gene polymorphism

## Abstract

**Background/aim:**

Sinonasal polyposis is a complex chronic disease displaying contributions from multiple genetic and environmental factors. In this study, we analyzed possible genetic factors that increase susceptibility to this widespread inflammatory disease.

**Materials and methods:**

A total of 176 adult patients, including 78 patients with sinonasal polyposis and 98 healthy controls, were analyzed for IL-1RN VNTR, IL-2(-330), and IL-4 VNTR gene polymorphisms using polymerase chain reaction and enzyme restriction.

**Results:**

IL-1RN and IL-4 VNTR polymorphisms were notably associated with sinonasal polyposis (P = 0.0001 and P = 0.036, respectively); however, regarding the IL-2(-330) gene polymorphism, no significant difference was shown between the patient and control groups (P = 0.235).

**Conclusions:**

Our study indicates that the RN2 allele of IL-1RN and the RP1 allele of IL-4 might be risk factors for developing sinonasal polyposis.

## 1. Introduction

Despite the fact that nasal polyposis was described for the first time by Hippocrates, its pathogenesis has yet to be clarified. Nasal polyposis is generally believed to involve a multitude of genetic and environmental factors; however, the exact mechanism that starts the ongoing inflammation is still debated. In the 2012 European Position Paper on Rhinosinusitis and Nasal Polyps (EPOS) documents, nasal polyposis was defined as a subgroup of chronic sinusitis (CRSwNP) [1]. Altered expression levels of cytokines and chemokines with proinflammatory, antiinflammatory, or angiogenic features are one of the hypotheses considered in the etiopathogenesis of sinonasal polyposis. 

A number of pro- and antiinflammatory cytokines are believed to play a role in the inflammatory response of sinonasal polyposis. Genetic polymorphisms have been considered important in the definition of the susceptibility profile for the development of this disease. The IL-1 family is a group of epithelial cytokines including the potent proinflammatory cytokines IL-1α and IL-1β; their negative regulator with an antiinflammatory effect is the IL-1 receptor antagonist (IL-1RN) [2]. The genes encoding the IL-1 family are located on the long arm of chromosome 2. The IL-1RN gene has a penta-allelic polymorphic site in the intron 2 region due to the presence of an 86-bp variable number of a tandem repeat (VNTR) sequence [3,4]. VNTR may result in genetic variations in an individual and may further alter the rate of gene transcription, the stability of mRNA, or the quantity and activity of the encoded protein. Individuals with different copy numbers of the repeat sequences differ in the number of potential protein binding sites, thereby altering the amount of cytokine production [5]. IL-1RN gene polymorphisms have been associated with the severity of or susceptibility to various inflammatory disorders [6].

IL-2 is a lymphokine produced by T cells and plays a major role in T and B cell cooperation. It induces the secretion of IL-1, interferon (IFN)-γ, and tumor necrosis factors (TNF)-α and TNF-β. IL-2 has powerful immunoregulatory effects on a variety of immune cells [7]. The gene coding for IL-2 is located on chromosome 4q26-q27 [8]. There is a functional T→G single nucleotide polymorphism (SNP) at position -330; it has been suggested that this polymorphism can be useful as a marker to diagnose susceptibility to various inflammatory diseases [9].

IL-4 is a prototypic member of Th2 cytokines and has potent antiinflammatory features. It reduces the production and impact of proinflammatory cytokines and is also involved in the isotype switching from immunoglobulin (Ig)M/IgG to IgE by B lymphocytes [10,11]. The IL-4 gene is located on chromosome 5q31–q33. The IL-4 variable number of tandem repeat (VNTR) polymorphism is characterized by a rare Rp1 allele (2 repeats = 183 bp), a frequent Rp2 allele (3 repeats = 253 bp), and Rp3 (4 repeats), which is a rarer allele.

In the present study, the association of IL-1RN VNTR, IL-2(-330), and IL-4 VNTR polymorphisms with sinonasal polyposis was evaluated. 

## 2. Materials and methods

### 2.1. Subjects

In this current study, we included 78 patients with nasal polyposis and 98 healthy controls with no history of nasal symptoms. The diagnosis of nasal polyposis was made by anterior rhinoscopy through various nasal endoscopes. The endoscopic equipment consisted of a series of various rigid rod lens Hopkins telescopes, a digital one-chip camera, a Xenon cold light source, and a high-resolution video monitor screen (all equipment was provided by Karl Storz Company, Tuttlingen, Germany). Computed tomography scans were performed to prevent any misdiagnoses. Patients with Samter’s triad or asthma were not included in this study. Our research was approved by the ethics committee of Adana City Education and Research Hospital. All patients and control subjects were informed about the study, and their written informed consents were obtained before commencing the study. Blood samples were collected in EDTA-coated vials and stored at –20 °C until required for genomic DNA extraction.

### 2.2. DNA extraction, oligonucleotide primers, PCR amplification, and restriction digestion

Genomic DNA was isolated from 250 μL of whole blood from each sample using proteinase K followed by an E.Z.N.A.® Tissue Kit II (Omega Bio-Tek, Norcross, GA, USA) in accordance with the manufacturer’s protocol. All DNA samples were examined at 260/280 nm absorbance ratio using a PeQLab Nanodrop (Biotechnologie GmbH, Rheinbreitbach, Germany). PCR amplifications were performed using 3 primer pairs (Table 1).

**Table 1:** Three primer pairs for amplification of IL-1RN, IL-2 (-330), and IL-4 gene polymorphisms.

Gene	Primers	
IL-1RN	F primerR primer	5′- ctcagcaacactcctat-3′ 5′- tcctggtctgcaggtaa-3′
IL2-330	F primerR primer	5′- tattcacatgttcagtgtagttct-3′5′- acattagcccacacttaggt-3′
IL-4	F primerR primer	5′- aggctgaaagggggaaagc-3′5′- ctgttcacctcaactgctcc-3′

Briefly, PCR was performed in a final volume of 25 μL containing 50 ng genomic DNA template, 10X PCR buffer with 50 Mm MgCl2, 100–500 nM of each primer, 10 μM dNTPs, and 5 U DNA polymerase. The DNA was initially denatured at 94 °C for 5 min prior to amplification. PCR amplification was accomplished using 35 cycles consisting of 30 s of denaturation at 94 °C, 45 s of annealing at 50 °C for IL-1RN, 58 °C for IL-4, and 52 °C for IL2-330 primers, and 30 s of extension at 72 °C, with a final extension cycle at 72 °C for 5 min. The PCR fragments of IL-1RA and IL-4 were separated using 1.5% agarose gel electrophoresis. 

IL-2-330 (rs2069762) SNP genotyping was performed using PCR-restriction fragment length polymorphism (PCR-RFLP). Restriction digestion was performed with PCR products of IL2-330 in a total volume of 10 μL amplicons, 2 μL 10X buffer “Tango”, and 10 units of FspBI (Bfal) enzyme. The samples were then incubated for 16 h at 37 °C; the digested PCR products were then separated using 3% agarose gel electrophoresis stained with ethidium bromide and visualized on a UV transilluminator with 50 base pair DNA ladder and photographed. After FspBI digestion, the IL2-330 products were separated into 3 fragments (A1, 150bp; A1/A2, 150, 127; and 23bp; A2, 127 and 23bp).

### 2.3. Statistics

SSPS v. 20 for Windows was applied for the statistical analysis of this study. The allele frequencies between the groups were analyzed by using the chi-squared test and Fisher’s exact test.

## 3. Results

This present study included a total of 176 subjects, 78 patients with nasal polyposis and 98 healthy controls. The ages of the nasal polyposis patients ranged from 15 to 80 years (mean: 49 years); 72% of the patient group was male and 28% was female. The age range of the control group was between 19 and 78 years (mean: 44 years). In the control group, 65% were male and 35% were female. This study genotyped the patient and control groups for the three polymorphisms.

**Figure 1:**
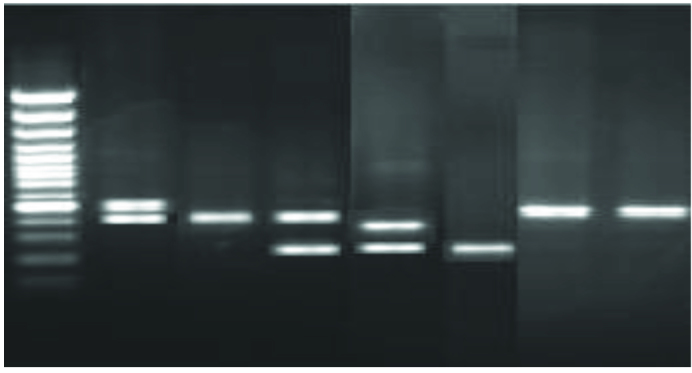
PCR amplification of genomic DNA analysis for rs 2234663; SNP of IL-1RN. Lane M: 100-bp DNA marker (Fermentas); lanes 1, 6, 7: 500-bp and 410-bp amplification fragments; lane 2: 410-bp amplification fragments; lane 3: 410-bp and 240-bp amplification fragments; lane 4: 325-bp and 240-bp amplification fragments; lane 5: 240-bp amplification fragments.

Genotypes for IL-1RN and IL-4 gene polymorphisms were analyzed in 78 samples in the study group. The IL-1RN allelic distribution showed RN1/RN1, RN1/RN2, RN1/RN3, RN2/RN2, and RN2/RN4 genotypes, and their frequencies were 48.7%, 38.46%, 2.56%, 8.97%, and 1.28%, respectively (Figure 1). 

**Figure 2:**
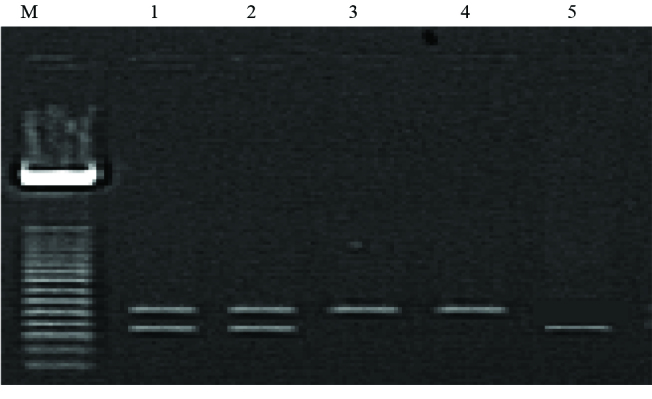
PCR amplification of genomic DNA analysis for rs 8179190; SNP of IL-4. Lane M: 50-bp DNA marker (Promega); lanes 1–2: 253-bp and 183-bp amplification fragments, lanes 3–4: 253-bp amplification fragment; lane 5: 183-bp amplification fragment.

The IL-4 allelic distribution showed RP1/RP1, RP1/RP2, and RP2/RP2 genotypes, and their frequencies were 5.12%, 26.92%, and 67.94%, respectively for each gene polymorphism (Figure 2).

**Table 2:** Distribution of genotypes frequencies of IL-1RA, IL-2 (-330), and IL-4 gene polymorphisms.

Gene Polymorphism	Reference SNP ID	Genotyping	Frequency	N (%)	p	Patients	Controls	
			IL-1RN	rs 2234663	RN1RN2RN3RN4	108 (69.23)45 (28.84)2 (1.28)1 (0.62)	172 (87.75)21 (10.71)1 (0.51)2 (1.02)	0.0001
IL-2-330	rs 206976	A1A2	95 (60.89)61 (39.10)	107 (54.59)89 (45.40)	0.235
IL-4	Rs 8179190	RP1RP2	29 (18.58)127 (81.41)	21 (10.71)175 (89.28)	0.036

**Figure 3:**
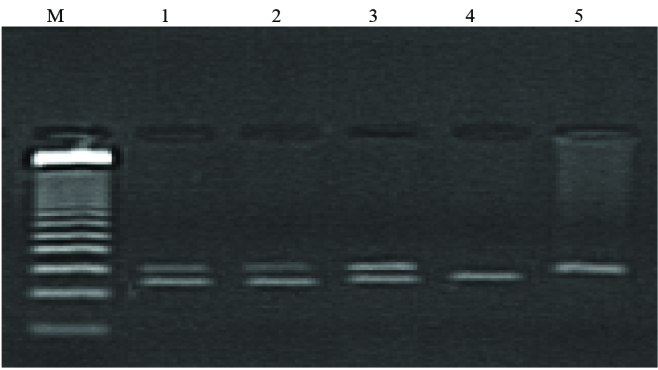
PCR-RFLP of genomic DNA analysis for rs 206976; SNP of IL-2(-330). Lane M: 50-bp DNA marker (Promega); lane 1–3: 150, 127, 23-bp fragments of the IL-2(-330) A1/A2 PCR product digested with FspBI; lane 4: 127-bp and 23-bp fragments A2/A2 PCR product digested with FspBI; lane 5: 150-bp and 23-bp fragments A1/A1 PCR product digested with FspBI.

Genotypes for IL-2(-330) gene polymorphisms were analyzed after restriction digestion on 78 samples in the study group (Figure 3).

The IL-2(-330) allelic distribution showed a preponderance of the homozygous dominant A1 alleles. The A1 allele occurred approximately three times more frequently in the study population than the A2 or mutant allele. Genotyping showed that the homozygous dominant genotype (A1A1) was also frequent in the study population, occurring at a frequency of 33.3%. The frequency of the heterozygote genotype (A1A2) was 55%; the mutant alleles (A2A2) were the least frequent, occurring at a frequency of only 11.66%. Distribution of genotypes frequencies of IL-1RA, IL-2 (-330) and IL-4 gene polymorphisms are shown in Table 2.

## 4. Discussion 

The present research is the first study to evaluate the role of IL-1RN, IL-2, and IL-4 gene polymorphisms in a Turkish population with nasal polyposis. Growing evidence implicates the contribution of genetic and environmental factors to the pathogenesis of chronic inflammatory diseases such as sinonasal polyposis. Our study has shown significantly higher incidence of RN2 and RP1 alleles in patients. IL-1 and IL-4 seem to play an important role in the pathogenesis of nasal polyposis because they activate and mobilize eosinophils and stimulate the differentiation and growth of B lymphocytes [12].

The IL-1 receptor antagonist gene polymorphism has been linked with a wide variety of diseases in the literature. Its association with preeclampsia, psoriasis, asthma, sepsis, inflammatory bowel disease, systemic lupus erythematosus, gastritis, schizophrenia, intellectual disability, cervical cancer, and even tonsillar hypertrophy has been studied recently [13–19]. The correlation between the IL-1 RN gene polymorphism and asthma has been demonstrated by various studies from Turkey, Egypt, South Africa, northern India, and Japan [20–23]. However, only one study has analyzed its relationship with chronic rhinosinusitis [24]. In the study of Cheng et al., where the study population included adult Taiwan-Chinese patients, they demonstrated that there was significant IL-1RN gene polymorphism in patients with CRSsNP, but no polymorphism in patients with CRSwNP. In our study, we demonstrated that the IL-1RN VNTR polymorphism was significantly associated with nasal polyps (P = 0.0001), and the RN2 allele of IL-1RN was a high risk factor for nasal polyp formation in the studied Turkish population.

Although it seems as though the findings of Cheng et al. conflict with those of our study, their study group only included an Asian population, which is genetically different from our Turkish study group of Caucasian origin. Zhang et al. presented the difference of cytokine patterns of nasal polyposis tissue of Asian patients [25]. In their research, they demonstrated that T(H)2 cytokine and related marker levels were significantly increased in the nasal polyposis tissue of white patients, whereas Asian patients showed a T(H)1/T(H)17 cell pattern, and high levels of IFN-γ, Th17, and neutrophil-related cytokines (IL-1β, IL-6, and IL-17). 

In this study, we also researched the IL-2(-330) gene polymorphism of patients with CRSwNP. This gene polymorphism is linked to many inflammatory and neoplastic diseases such as basal cell carcinoma, Graves’ disease (IL-2 -330T/G), hepatocellular carcinoma; multiple sclerosis IL-2 (-330 T/T), Behçet’s disease where the IL-2 (-330 GT) genotype shows susceptibility and the IL-2(-330 T/T) genotype shows a preventive impact, nasopharyngeal carcinoma IL-2 (-330 T/G), neuroendocrine tumors, and asthma IL-2 (-330) and IL-2 (+166) [26–33]. The gene and haplotype profiles of patients with asthma and chronic obstructive airway disease (COPD) have been mentioned in a few studies. Trajkov et al. demonstrated a positive association between patients with COPD and the IL-2(-330/T:T) genotype and IL-2/TG haplotype, and a negative association with the IL-2(-330/G:T) genotype [34]. In contrast, Movahedi et al. reported a positive correlation with IL-2 GT at position -330 and a negative correlation with IL-2 TT at position -330 in patients with asthma [35]. Although the reports were conflicting, they showed a possible relationship between the IL-2 330 gene polymorphism and inflammatory lung diseases. Additionally, Hamilos et al. demonstrated that IL-2 mRNA expression was significantly higher in allergic and aspirin-tolerant CRSwNP, and also found that tissue eosinophilia and T lymphocyte infiltration was not related with IL-2 mRNA expression [36]. We could not find any research considering the linkage between the IL-2(-330) gene polymorphism and CRSwNP in the current literature, and we found no significant relationship between them in our study.

IL-4, a Th2 cytokine with an antiinflammatory effect, is one of the most studied cytokines of CRSwNP. The increased IL-4 level in nasal polyposis has been reported in several studies [37]. Milonski et al. demonstrated increased IL-4 gene expression in atopic patients with CRSwNP [38]. Murowicka et al. showed that the C/T polymorphism of the IL4 gene was not associated with nasal polyp formation [39]. Zhang et al. showed that the IL-4 polymorphism of 33T>C and -590C>T were positively linked with susceptibility to CRS [40]. Park et al. showed that the IL-4 promoter polymorphism of -590 C/C was linked with the increased expression of 5-LO in patients with CRSwNP [41]. Yea et al. from Korea demonstrated that the IL-4 C-590T polymorphism was protective against nasal polyp formation [42]. In our study, we searched for IL-4 VNTR gene polymorphisms in CRSwNP. IL-4 VNTR gene polymorphisms have been the subject of various studies from Turkey. Their association with coronary artery disease, knee osteoarthritis, alopecia areata, diabetic peripheral neuropathy, recurrent aphthous stomatitis (RAS), multiple sclerosis, and Behçet’s disease in Turkish population have all been studied previously [43–49]. IL-4 VNTR gene polymorphisms have also been linked with immune thrombocytopenic purpura (ITP) and asthma in the current literature [50,51]. In addition, IL-4 and IL-1RN (VNTR) gene polymorphisms have together been linked to frailty syndrome and type 2 diabetes mellitus [52,53]. In our research, we found that IL-4 VNTR polymorphisms were significantly associated with nasal polyps in the Turkish population (P = 0.036). Our study shows the effect of the RP1 allele of IL-4 on the susceptibility of polyposis formation in patients with CRSwNP. 

In conclusion, the etiopathogenesis of sinonasal polyposis is still an unsolved puzzle of otorhinolaryngology. The theories that try to enlighten the exact mechanism for the chronic inflammation become more complex every day. In this study, we researched possible gene polymorphism associations and found that the RN2 allele of IL-1RN and the RP1 allele of IL-4 were high risk factors for developing sinonasal polyposis in the studied Turkish population. To our knowledge, this is the first case-control study from Turkey to analyze the impact of IL-1RN VNTR, IL-2(-330), and IL-4 VNTR polymorphisms in the pathogenesis of sinonasal polyposis. Further studies are needed to demonstrate the role of these gene polymorphisms in larger patient groups, as our study group was limited.
